# Fragmentation of metal(II) bis(acetylacetonate) complexes induced by slow electrons

**DOI:** 10.3762/bjnano.14.81

**Published:** 2023-09-26

**Authors:** Janina Kopyra, Hassan Abdoul-Carime

**Affiliations:** 1 Siedlce University of Natural Sciences and Humanities, Faculty of Sciences, 3 Maja 54, 08-110 Siedlce, Poland; 2 Universite de Lyon, Université Lyon 1, Institut de Physique Nucléaire de Lyon, CNRS/IN2P3, UMR5822, F-69003 Lyon, France

**Keywords:** dissociative electron attachment, gas phase, metal(II) bis(acetylacetonate), negative ions, organometallic complexes

## Abstract

Nowadays, organometallic complexes receive particular attention because of their use in the design of pure nanoscale metal structures. In the present work, we present results obtained from a series of studies on the degradation of metal(II) bis(acetylacetonate)s induced by low-energy electrons. These slow particles induce the formation of the acetylacetonate anion, [acac]^−^, and the parent anion as the most dominant species at incident electron energies near 0 eV. They also fragment the organometallic compounds via various competitive reaction channels that occur at higher energies via dissociative electron attachment. The reported data may contribute to a better understanding of the physical chemistry underlying the electron–molecule interactions, which is crucial for potential applications of these molecular systems in the deposition of nanoscale structures.

## Introduction

Nowadays, organometallic compounds are used in many applications. They receive great interest in the field of nanoscale technologies [1–3]. For example, in nanoscale design processes, the combination of an electron beam with an organometallic target (e.g., focused electron beam-induced deposition, FEBID) is a promising technique for direct 3D deposition of high-purity materials with minimum residual carbon in the product on the surface [4–5]. The FEBID precursor molecules adsorb and diffuse on the surface, where they are decomposed under the focused kiloelectronvolt electron beam. In this technique, both the primary ionizing particles and the secondary species (e.g., ballistic electrons) with energies below 20 eV [6–7] lead to the decomposition of the molecules and the subsequent surface modification. The general mechanisms of the different involved processes have been reviewed regarding the energy range of the irradiating particles [8]. The collision of high-energy electrons with molecules leads to ionization and fragmentation. At energies near the molecular ionization energies and below, the fragmentation arises from a resonant mechanism known as dissociative electron attachment (DEA) producing exclusively a negative fragment ion and one or more neutral counterparts, as it will be discussed below. The contribution of each of the processes may depend on the nature of the organometallic precursors.

Metal bis(acetylacetonate) complexes are of interest for many thin film fabrication techniques (e.g., chemical vapor deposition [9], atomic layer epitaxy [10], or atomic layer etching [11]) and as precursors for carbon materials, such as carbon nanotubes and carbon onion particles [12], or metal oxide nanocrystals [13–14]. The popularity of these compounds is related to their volatile nature, ease of preparation, and often lower air sensitivity and toxicity in comparison to organometallic compounds containing carbon–metal bonds (e.g., metallocenes). In the context of a potential use of these organometallic complexes, it is desirable to investigate the physical chemistry, in particular, the processes induced by the interaction of these molecular systems with low-energy electrons.

We performed a series of collision experiments of low-energy electrons with metal bis(acetylacetonate)s, ML_2_, where M and L represent a metal (Mn, Co, Ni, Cu, and Zn) and the acetylacetonate ligand, respectively. Previous reports were only dedicated to the mechanism and the energetics for the production of the two predominant species, that is, the parent [ML_2_]^−^ anion and the ligand [L]^−^ anion [15–19]. In addition to these two species, the interaction of slow electrons with the metal chelates also produces a rich variety of fragment anions, which are reported and discussed in the present report. A comprehensive picture of the fragmentation pattern of each ML_2_ precursor, including the quantification of dissociation pathways (e.g., branching ratio), will be helpful for using this family of organometallic compounds.

## Results and Discussion

**Figure 1:**
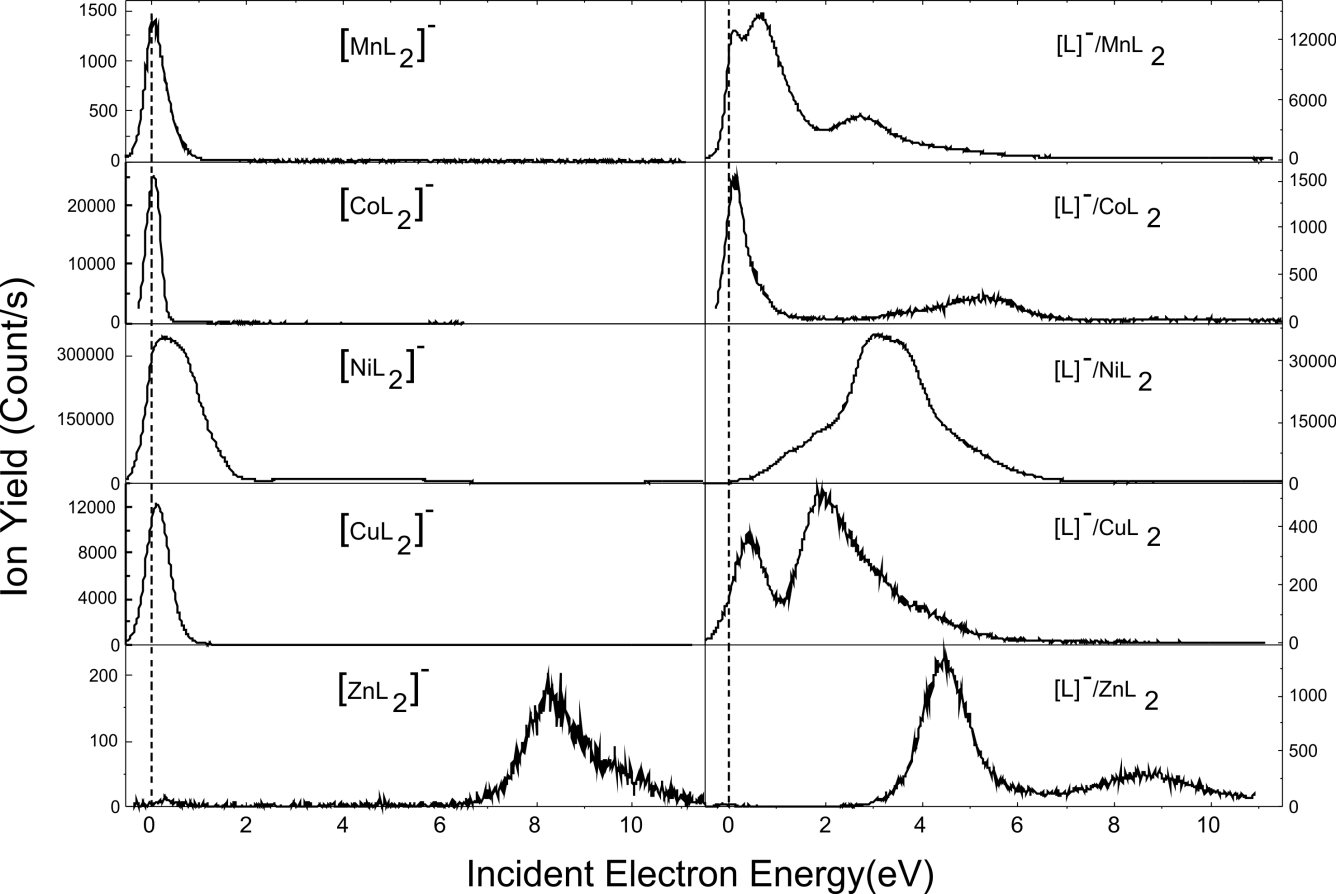
[ML_2_]^−^ and [L]^−^ yield functions measured from the interaction of electrons with thermally evaporated metal(II) bis(acetylacetonate) (M: Mn, Co, Ni, Cu, and Zn). Data for [ML_2_]^−^ and [L]^−^ are from [15–19].

**Figure 2:**
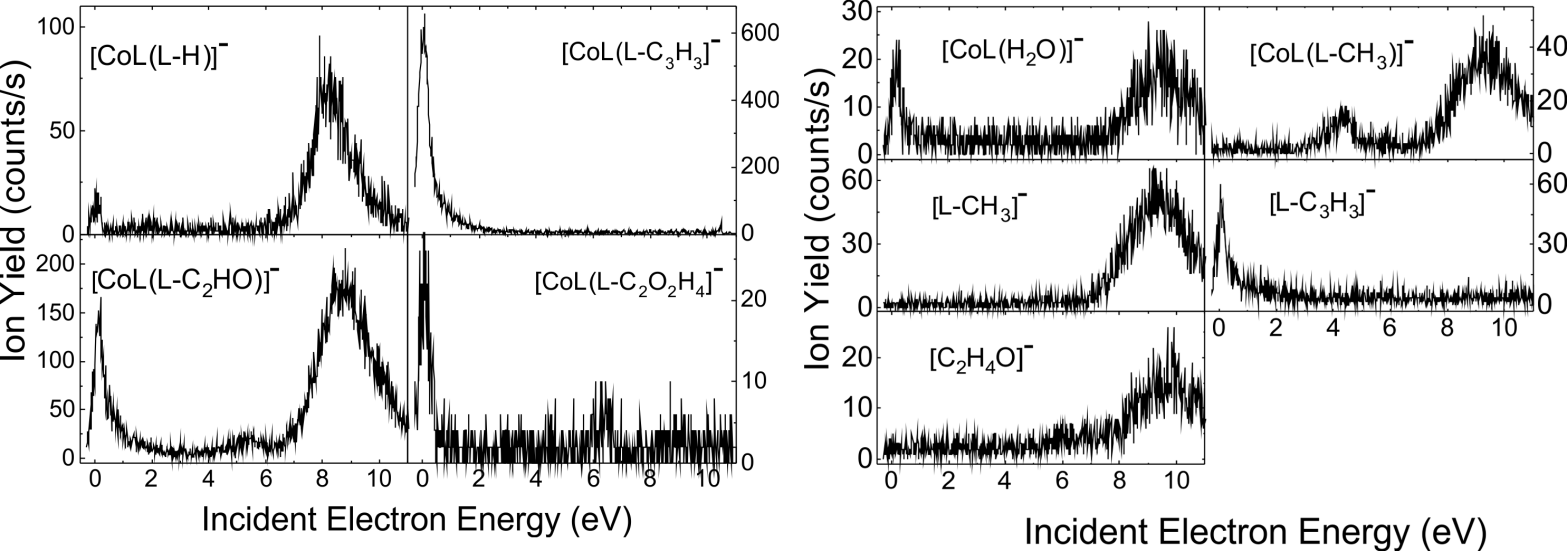
Yield of the fragment anions [CoL(L-H)]^−^, [CoL(L-C_3_H_3_]^−^, [CoL(L-C_2_HO]^−^, [CoL(L-C_2_O_2_H_4_]^−^, [CoL(H_2_O)]^−^, [CoL(L-CH_3_]^−^, [L-CH_3_]^−^, [L-C_3_H_3_]^−^, and [C_2_H_4_O]^−^ obtained from DEA of Co(acac)_2_.

**Figure 3:**
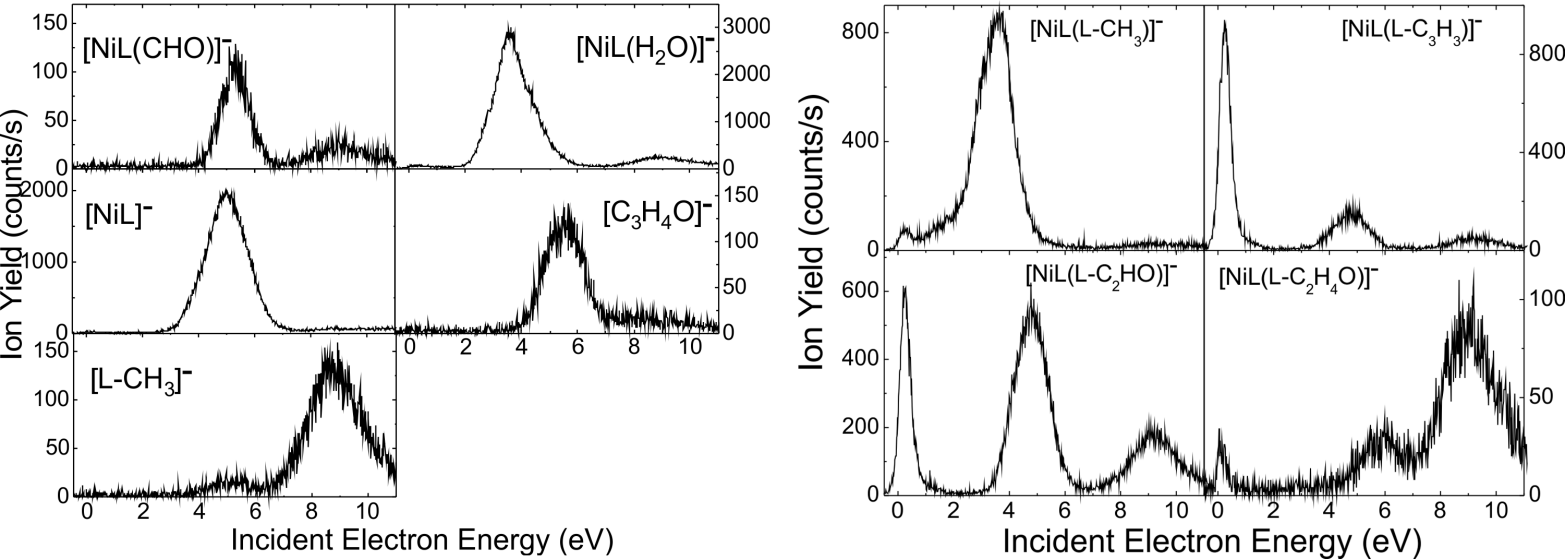
Yield of the fragment anions [NiL(CHO)]^−^, [NiL(H_2_O)]^−^, [NiL]^−^, [C_3_H_4_O]^−^, [L-CH_3_]^−^, [NiL(L-CH_3_]^−^, [NiL(L-C_3_H_3_]^−^, [NiL(L-C_2_HO]^−^, and [NiL(L-C_2_H_4_O]^−^ obtained from DEA of Ni(acac)_2_.

**Figure 4:**
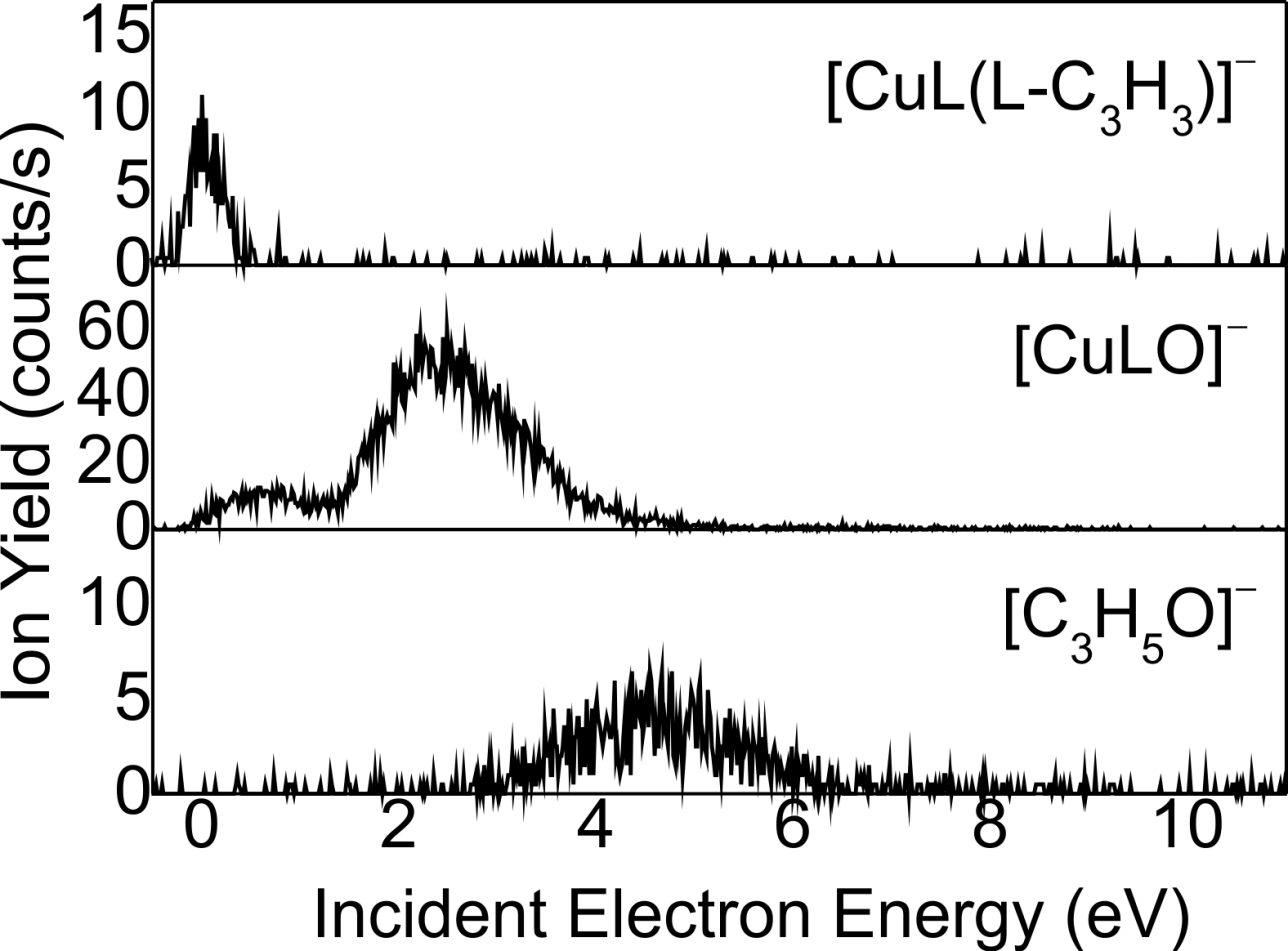
Yield of the fragment anions [CuL(L-C_3_H_3_]^−^, [CuLO]^−^, and [C_3_H_5_O]^−^ obtained from DEA of Cu(acac)_2_. Data for the ion yields of [CuL(L-C_3_H_3_]^−^, [CuLO]^−^, and [C_3_H_5_O]^−^ were taken from [15].

**Figure 5:**
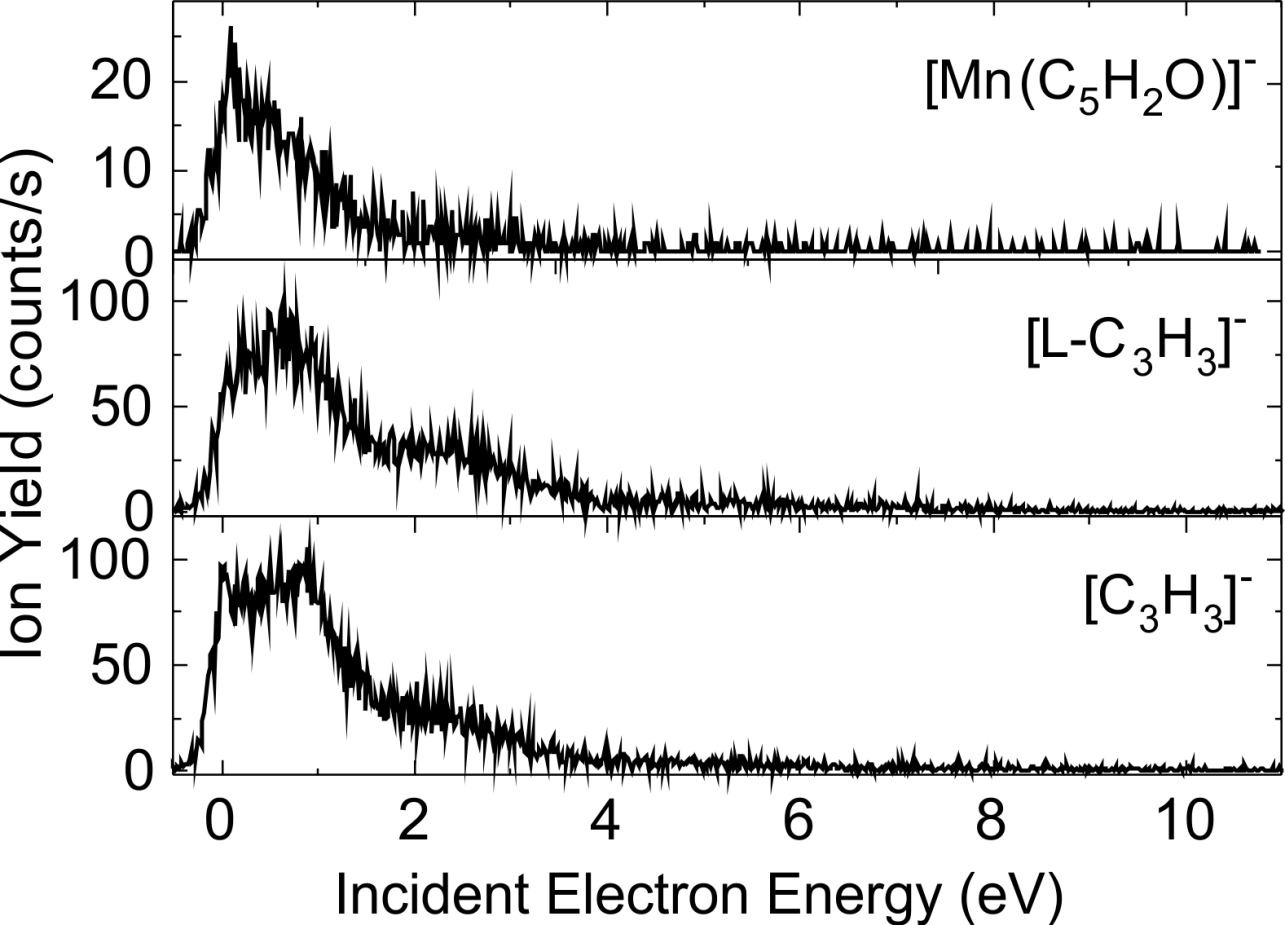
Yield of the fragment anions [Mn(C_5_H_2_O)]^−^, [L-C_3_H_3_]^−^, and [C_3_H_3_]^−^ obtained from DEA of Mn(acac)_2_.

**Figure 6:**
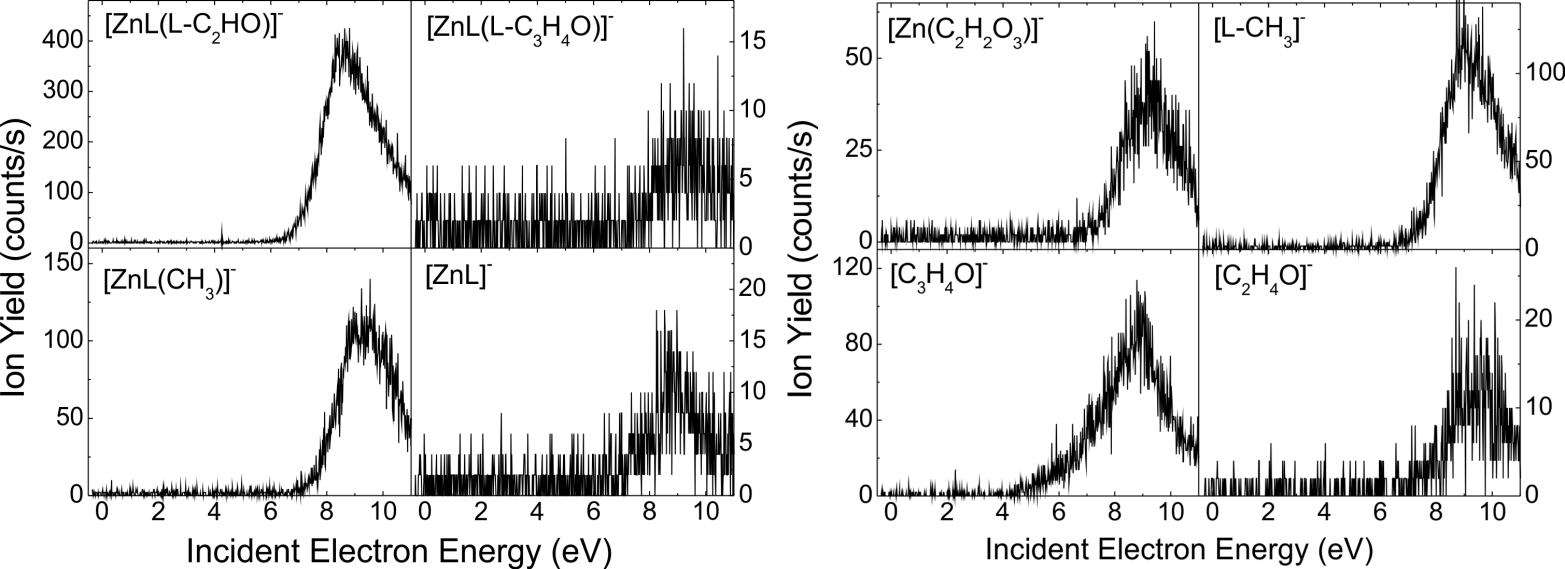
Yield of the fragment anions [ZnL(L-C_2_HO]^−^, [ZnL(L-C_3_H_4_O)]^−^, [ZnL(CH_3_)]^−^, [ZnL]^−^, [Zn(C_2_H_2_O_3_)]^−^, [L-CH_3_]^−^, [C_3_H_4_O]^−^, and [C_2_H_4_O]^−^ obtained from DEA to Zn(acac)_2_.

The interaction of low-energy electrons with gaseous compounds ML_2_ (M: Mn, Co, Ni, Cu, and Zn; L: acac) produces the parent anion [ML_2_]^−^ and the fragment anion [L]^−^ as the predominant species (Figure 1). Further fragment anions were observed and they are shown below in Figures 2–6. The Ni, Co, and Zn complexes yield a larger number of anions (eleven anions from Ni and Co complexes and ten from the Zn-containing compound) than the other investigated organometallic compounds. Table 1 summarizes all the observed anion products. As the anion yields shown below in Figures 1–6 show structures characteristic of resonant mechanisms, Table 1 also reports the peak positions of the fragment anions and their branching ratios derived by integration of the ion yield over the respective peaks.

**Table 1:** Energies (eV) of the peak maximum obtained from Figures 1–6 (see below). In parentheses, the fragmentation branching ratios (%) calculated by integrating the yield of the fragment anions at the peak position are provided. The asterisks indicate peaks with weak intensity.

	MnL_2_	CoL_2_	NiL_2_	CuL_2_	ZnL_2_

[ML_2_]^−^	ca. 0.1	ca. 0.1	0.25 0.65 4.5	0.1	8.1 (18.60) 8.9
[ML(L-H)]^−^		0.1 8.3 (5.0)			
[ML(L-CH_3_)]^−^		4.4 9.3	0.2 ca. 1.3 2.9 3.6 (1.7)		
[ML(L-C_3_H_3_)]^−^		0.4	0.2 4.8 (1.0) 9.2 (4.3)	0.1	
[ML(L-C_2_HO)]^−^		0.2 5.4 (12.1) 8.8 (53.8) 10.0*	0.2 4.8 (4.1) 9.2 (21.6)		8.4 (20.07) 9.5
[ML(L-C_2_H_4_O)]^−^			0.1 5.9 9.0 (20.2)		
[ML(L-C_3_H_4_O)]^−^					ca. 9.4*
[ML(L-C_2_H_4_O_2_)]^−^		0.2 6.4*			
[ML(CHO)]^−^			5.3 (0.7) 9 (3.7)		
[ML(H_2_O)]^−^		0.2 9.3 (4.4)	ca. 0.1 3.6 (5.0) 8.9 (32.0)		
[MLCH_3_]^−^					9.4
[MLO]^−^				0.6 2.5 (44.3)	
[ML]^−^			5.0 (19.1)		9.0*
[M(C_5_H_2_O)]^−^	ca. 0.1 0.5 (0.12)				
[M(C_2_H_2_O_3_)]^−^					9.3
[L]^−^	ca. 0.0 0.62 (98.64) 2.7 (98.80)	0.2 0.7 3.8 5.1 (85.5)	1.4 1.8 2.8 3.6 (93.3) 4.9 (73.9)	0.4 1.8 2.6 (55.7) 3.5	4.2 4.8 8.4 (61.33)
[L-CH_3_]^−^		9.3 (25.7)	ca. 5.1* (<0.01) 8.7		9.3
[L-C_3_H_3_]^−^	0.1 0.7 (0.60) 2.1 (0.64)	0.1			
[C_3_H_5_O]^−^				4.6	
[C_3_H_4_O]^−^			5.5 (1.0)		8.8
[C_2_H_4_O]^−^		9.6 (5.5)			9.5*
[C_3_H_3_]^−^	0.1 0.7 (0.64) 2.1 (0.56)				

At the electron energy range investigated in the present work, that is, below 10 eV, DEA, as well as neutral dissociation and dissociative ionization, are the mechanisms responsible for the efficient fragmentation of molecules. In the case of dissociative electron attachment, studied in the present work, the incoming scattering electron is captured by the precursor molecule to form a transient negative ion, TNI or [ML_2_]^#−^. If the electron autodetachment time of the TNI is longer than the dissociation time, the transient anion undergoes dissociation into a negative fragment and one or more neutral counterpart(s). The precursor anion may also survive for a sufficiently long time, that is, before the electron autodetachment process takes place, through the fast redistribution of the excess energy carried by the attaching electron. The formation of TNIs follows different mechanisms. Briefly, the electron may be trapped into a usually unfilled molecular valence orbital (i.e., shape resonance) [20]. Such a process usually arises typically at electron energies below 4 eV. The molecular orbitals into which the excess electron may be trapped are reported in [15–19] for the investigated organometallic systems. Alternatively, the electron can also be attached to an electronically excited state resulting in core-excited or Feshbach resonances [21]. This process takes place at energies above the first electronically excited state of the molecule, that is, typically at ca. 4 eV for most organic molecules. However, for molecules that contain metal atoms, this energy can be as low as 1.3–1.4 eV [17]. The formation of the [ML_2_]^−^ anion via the core-excited resonance occurs in NiL_2_ and ZnL_2_ at around 5 and 8 eV, respectively (Figure 1). Such “high”-energy resonances are unusual; however, they have been observed for large molecules, such as phthalocyanine or tetraphenylporphyrin (up to 7–8 eV), because of the long lifetime of the TNIs [22]. Also, the electron can be trapped by long-range forces to form a “multipole”-bound anion. This bound state may then couple to some dissociative valence states, leading to TNI decomposition [23]. ML_2_ compounds usually possess high dipole moment, quadrupole moment, and/or polarizability [15–19], which allow this mechanism to occur. All these processes are involved in the yield functions reported below in Figures 1–6.

At first glance, the formation of the precursor anion is very effective for the Co-, Ni- and Cu-containing organometallic complexes, in contrast to MnL_2_ and ZnL_2_ (Figure 1). These experimental observations may be related in part to the adiabatic electron affinity values of the neutral precursors, which have been calculated to be 1.456, 1.124, 0.498, 0.44, and 0.16 eV for CuL_2_, NiL_2_, and CoL_2_, MnL_2_, and ZnL_2_, respectively [15–19]. As the molecules are sublimated, the pressure of the molecular beam, *P*, that is, the number of precursors in the gas phase, follows the Clausius–Clapeyron relation (log(*P*) = *C* − Δ*H*_sub_/*T*), where *C* is a constant. Taking into account the sublimation enthalpy, Δ*H*_sub_, for MnL_2_ and CoL_2_ (i.e., 139.3 and 130.1 kJ/mol, respectively [24]), and the working temperature (i.e., 390 and 420 K, respectively), we estimate the value of *P*_CoL2_/*P*_MnL2_ to be about 38, assuming *C* to be constant. This ratio of pressures, also representing the ratio of the density of neutral precursors, must then be weighted with the (yet unknown) electron attachment cross sections to compare the experimentally measured ratio between CoL_2_^−^ anions and MnL_2_^−^ anions, via *N*_ion_ = ε·*N*_e_·*N*_neutral_·σ_ion_·*L*, where ε is the ion detection efficiency, *N*_e_ and *N*_neutral_ are the number of colliding electrons (intensity of the electron current) and the density of the neutral precursor targets, respectively, *L* is the collision length, and σ_ion_ is the cross section for the ion production. The experimental estimate of *N*_CoL2−_/*N*_MnL2−_ from Figure 1 is around 15. This result suggests that under the same experimental conditions (sublimation temperatures), the production of [ML_2_]^−^ is comparable.

The stabilization of the precursor anion, [ML_2_]^−^, observed for electron attachment to copper hexafluoroacetylacetone, has been suggested to arise from the isomerization from the planar to the tetrahedral configuration [25]. This change of geometry has been calculated for CuL_2_, for which one of the ligands rotates by 90° from the planar configuration [15]. Note that the planar anion may also co-exist, but this configuration is unstable [15]. Surprisingly from the DFT calculations, the neutral structure of ML_2_ (M: Mn, Ni, Co, and Zn) exhibits the same configuration as the stable anion [ML_2_]^−^ [16–19]. According to DFT calculations, the [ML_2_]^−^ anion configuration is lower in energy than the neutral configuration [15–19]. It has also been shown that the metal–ligand M···O bond length increases substantially for Co, Ni, and Cu organometallic complexes when the equilibrium configuration of neutral precursor molecule is changed to the anion configuration. For example, for NiL_2_, this distance changes from 1.86 to 2.026 Å [18]. As a consequence, the stabilization of the precursor anion leads to a substantial increase in the M···O bond length. Thus, stabilization of the [ML_2_]^−^ anion prevents the dissociation of the TNI and, hence, the production of [L]^−^ near 0 eV. Furthermore, the formation enthalpy of this fragment has been calculated to be 2.01, 1.72, 0.95, 1.54, and 1.74 eV for Mn, Co, Ni, Cu, and Zn complexes, respectively [15–19]. Hence, these anion species may be generated at energies higher than the values given above. It should be noted that the metal acetylacetonates crystalize in a trimeric form. Therefore, in thermal evaporation experiments below the decomposition temperatures of the material, ML_3_, in which the metal is coordinated by three ligands [26–27], may also be produced at least to a small extent at the studied temperatures. Indeed, it has been observed that thermal desorption of solid thymine produces not only the monomer, but also some small contribution of the dimer of the nucleobase [28]. Thus, we have suggested that the production of the [L]^−^ anion near 0 eV may occur from the scattering of electrons at trimeric complexes for which the formation enthalpies have been calculated to be exothermic [15–19].

As shown in Table 1, the fragmentation of the transient [ML_2_]^#−^ anion results in a wide variety of negatively charged species. They arise from the decomposition of one of the ligands into X species, whereas the second ligand still binds to the metal, for example, [MLX]. The loss of C_3_H_3_ from one of the ligands, that is, (L-C_3_H_3_), is observed for CoL_2_ (Figure 2), NiL_2_ (Figure 3), and CuL_2_ (Figure 4), as [ML(L-C_3_H_3_)]^−^ anion (Table 1).

We can notice that the Mn (Figure 5) and Cu (Figure 4) complexes produce fewer fragments than those containing cobalt, nickel, and zinc. Furthermore, the fragmentation of the two complexes occurs mainly at electron energies below 4 eV. The decomposition of [ML_2_]^#−^ also results in the localization of the excess charge on one of the ligands, that is, the [L]^−^ anion, or on the decomposition product of the ligand, for example, the negative [C_3_H_4_O]^−^ species observed for complexes containing Ni (Figure 3) and Zn (Figure 6). The [L]^−^ anions are, in general, produced at an appreciably high rate, whereas the complementary fragment, [ML]^−^, is either observed with a much lower intensity (i.e., for Ni (Figure 3) and Zn (Figure 6)) or not detected at all under the present experimental conditions. Finally, it is interesting to note that the fragmentation of ML_2_ arises exclusively at electron energies below 4 eV, with the exception of ZnL_2_, for which the formation of the parent anion is observed at electron energies above 6 eV.

For each of the organometallic complexes, Table 1 reports the energy of the maximum of the peaks associated with the production of a specific parent or fragment anions. It is noteworthy that different fragments are generated at the same incident electron energy, indicative of competitive fragmentation channels. When the integrated yields at this energy are compared with the sum of all integrated yields produced, we can provide the branching ratios for fragment production. The [MnL_2_]^#−^ TNI decomposes into the negative fragments [L]^−^, [L-C_3_H_3_]^−^, and [C_3_H_3_]^−^ at ca. 2.6 eV (Figure 5). For instance, at this energy, we estimate the branching ratios for the [L]^−^, [L-C_3_H_3_]^−^, and [C_3_H_3_]^−^ anionic fragments to be 98.80%, 0.64%, and 0.56%, respectively. It is noteworthy that the decomposition channels for the production of the fragment anions [L-C_3_H_3_]^−^ and [C_3_H_3_]^−^ are comparable, but their yields are much lower than the yield of the [L]^−^ anion. The NiL_2_ complex exhibits many competitive fragmentation pathways at an energy of ca. 5 eV. At this energy, the channel leading to [L]^−^ represents 73.9% while that producing the [NiL]^−^ fragment anion is estimated to be 19.1% (Figure 3). The sum yield of the other channels represents approximately 7.0% of the TNI decomposition. As the resonance energy provides information on the electron capture process, the branching ratio provides information on the dynamics of the decay channels of the TNI.

## Conclusion

The interaction of low-energy electrons with metal(II) bis(acetylacetonate) complexes produces mainly the parent anion [ML_2_]^−^ and the fragment anion [L]^−^. The stabilization of the precursor anion near a threshold energy leads to substantial changes in the metal–ligand M···O bond length, with or without a change in the configuration of the neutral precursor. In addition, a large variety of anionic species can be detected; however, they are produced with small yields. They arise from the decomposition of the ligand, with the excess charge residing on an organic fragment, either the one that contains or the one that does not contain the metal. Some of these fragment anions are generated via competitive fragmentation channels at specific electron energies, indicative of the decay of the same transient negative ion. It is noteworthy that the Mn- and Cu-containing organometallic complexes produce fewer fragments, which arise almost exclusively at electron energies below 4 eV. Characterization of the dissociation products and the pathways for anion production will contribute to a better understanding of the fundamental chemistry of ML_2_ compounds. This is desirable for potential applications requiring the knowledge of decomposition of such organometallic complexes by particles (e.g., electron beams), in plasma applications, or possibly in radiation therapy as radiosensitizers.

## Experimental

We performed electron collision experiments with several metal acetylacetonate compounds, ML_2_, in a crossed-beam arrangement. According to the description given in [16–17,29], this arrangement consists of an electron source, an oven, and a quadrupole mass analyzer (QMA). The components are housed in a UHV chamber at a base pressure of around 2 × 10^−8^ mbar. A well-defined electron beam generated from a trochoidal electron monochromator (resolution approx. 210 meV FWHM), orthogonally intersects an effusive molecular beam of ML_2_. The latter emanates from a vessel containing approximately 1 mg of ≥98% purity powder (products purchased from Alfa Aesar) heated by two in vacuo halogen bulbs. The materials were used as received without further purification. The lamps also maintain all of the electrostatic lenses and plates at oven temperature to prevent powder condensation, which otherwise may lead to undesirable changes in contact potentials during the measurements. The present experiments were carried out at temperatures typically 10–20 K below the temperatures for which the decomposition of the molecules has been observed from thermochemistry methods [30–34]. Thus, the vessel temperatures in these experiments were 390, 420, 425, 400, and 375 K for MnL_2_, CoL_2_, NiL_2_, CuL_2_, and ZnL_2_, respectively. At these temperatures, it is very likely that the compounds are evaporated intact for collision experiments. Negative ions that are produced in the reaction area after the electron–molecule interaction are extracted from the collision area by a small draw-out field (ca. 0.5 V·cm^−1^), then analyzed by the QMA and finally detected using a single-pulse counting technique. The electron energy scale was calibrated using a flow of SF_6_ gas through the oven that produced the well-known SF_6_^−^ resonance near 0 eV. The measurements were performed without the presence of the calibration gas, avoiding potentially unwanted reactions such as dissociative electron transfer with the investigated molecules producing an additional signal near 0 eV [35].
